# Visual and quantitative assessment of hip implant-related metal artifacts at low field MRI: a phantom study comparing a 0.55-T system with 1.5-T and 3-T systems

**DOI:** 10.1186/s41747-023-00320-5

**Published:** 2023-02-08

**Authors:** Hanns-Christian Breit, Jan Vosshenrich, Martin Clauss, Thomas J. Weikert, Bram Stieltjes, Balázs K. Kovacs, Michael Bach, Dorothee Harder

**Affiliations:** 1 Department of Radiology, University Hospital Basel, Petersgraben 4, 4031 Basel, Switzerland; 2 Department of Orthopaedics and Traumatology, |University Hospital Basel, Spitalstrasse 21, 4031 Basel, Switzerland

**Keywords:** Artifacts, Titanium, Hip prosthesis, Image processing (computer-assisted), Magnetic resonance imaging

## Abstract

**Background:**

To investigate hip implant-related metal artifacts on a 0.55-T system compared with 1.5-T and 3-T systems.

**Methods:**

Total hip arthroplasty made of three different alloys were evaluated in a water phantom at 0.55, 1.5, and 3 T using routine protocols. Visually assessment (VA) was performed by three readers using a Likert scale from 0 (no artifacts) to 6 (extremely severe artifacts). Quantitative assessment (QA) was performed using the coefficient of variation (CoV) and the fraction of voxels within a threshold of the mean signal intensity compared to an automatically defined region of interest (FVwT). Agreement was evaluated using intra/inter-class correlation coefficient (ICC).

**Results:**

Interreader agreement of VA was strong-to-moderate (ICC 0.74−0.82). At all field strengths (0.55-T/1.5-T/3-T), artifacts were assigned a lower score for titanium (Ti) alloys (2.44/2.9/2.7) than for stainless steel (Fe-Cr) (4.1/3.9/5.1) and cobalt-chromium (Co-Cr) alloys (4.1/4.1/5.2) (*p* < 0.001 for both). Artifacts were lower for 0.55-T and 1.5-T than for 3-T systems, for all implants (*p* ≤ 0.049). A strong VA-to-QA correlation was found (*r* = 0.81; *p* < 0.001); CoV was lower for Ti alloys than for Fe-Cr and Co-Cr alloys at all field strengths. The FVwT showed a negative correlation with VA (-0.68 < *r* < -0.84; *p* < 0.001).

**Conclusions:**

Artifact intensity was lowest for Ti alloys at 0.55 T. For other alloys, it was similar at 0.55 T and 1.5 T, higher at 3 T. Despite an inferior gradient system and a larger bore width, the 0.55-T system showed the same artifact intensity of the 1.5-T system.

## Key points

- Low field magnetic resonance imaging (MRI) currently experiences a renaissance.
- Low field (0.55 T) MRI resulted to be equal to 1.5-T MRI in terms of artifact reduction, even with a weaker gradient system and larger bore size.
- 3-T MRI resulted to be inferior to 1.5-T and 0.55-T MRI in terms of artifact reduction for standard alloys hip implants

## Background

Worldwide demand for total hip arthroplasty continuously increases and is projected to multiply within the next years [1, 2]. Especially in younger patients of less than 65 years of age, this trend will probably cause a similar rise in revision surgery, given artificial hips’ limited lifetime [3, 4]. As a consequence, also the need for magnetic resonance imaging (MRI) in this population, *e.g.*, to assess complications such as implant failure or loosening [5] as well as pathologies in the surrounding soft tissues [6], will likely increase to a certain extent.

The diagnostic capabilities of MRI have been proven to be superior to ultrasound and computed tomography in this setting, especially when aiming to detect and quantify muscle atrophy, pseudotumors, and other soft tissue changes [7–9]. This further emphasizes the key role of MRI in future imaging musculoskeletal diagnostic pathways.

Traditionally, MRI of metal implants has been challenging due to susceptibility-induced artifacts, degrading image quality and diagnostic accuracy. Extensive research in the fields of protocol optimization and metal artifact reduction techniques over the last decades led to strategies to improve image quality. Two of these techniques are slice encoding for metal artifact correction (SEMAC) and view angle tilting (VAT). They proved to reduce artifacts, however at the cost of prolonging scanning times [10, 11]. Since artifact severity is highly dependent on implant alloys, these techniques are somehow limited in their capabilities [12]. A further promising approach to reduce susceptibility artifact-related image degradation might be the use of MRI systems with lower field strengths. However, data on susceptibility artifacts in low-field MRI below 1.0-T field strength is limited [13].

Finally, the impact of implant-related artifacts on diagnostic accuracy and clinical decision making might differ between radiologists and orthopedic surgeons who review images for preoperative planning. Assessment of artifact severity and image quality thus remains challenging and is performed either qualitatively [11, 14] or quantitatively [15].

The aim of our study was to visually and quantitatively investigate hip implant-related metal artifacts of three distinct implant alloys on a newly introduced 0.55-T scanner system in comparison to images acquired on 1.5-T and 3-T systems with metal artifact reduction sequences (MARS) from clinical routine.

## Methods

Approval by the institutional review board as well as informed consent was not required for this prospective experimental phantom study.

### Phantom

**Fig. 1:**
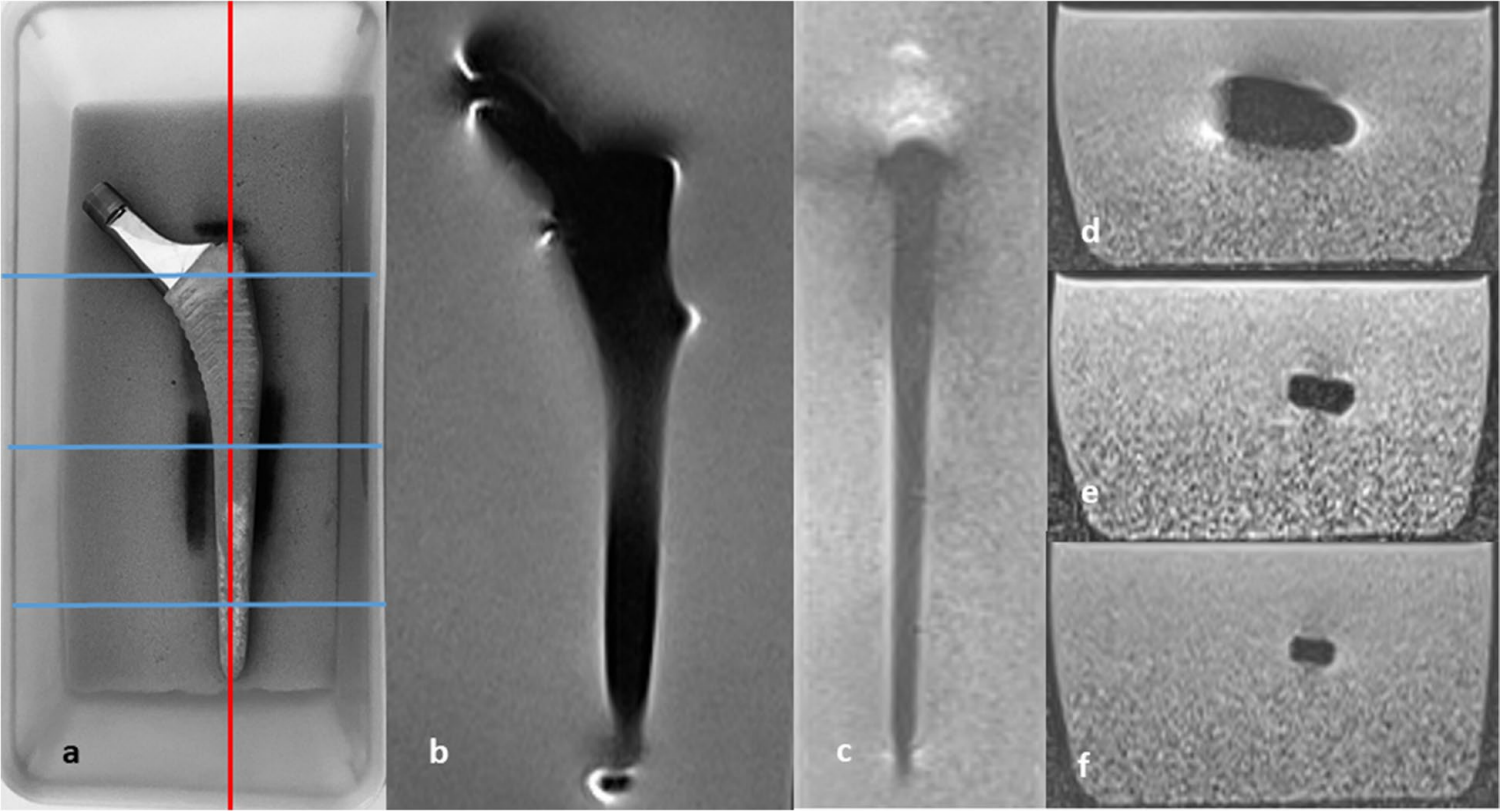
Femoral component on a foam pad within a water phantom (**a**). Corresponding slice positions in coronal (**b**), sagittal (**c**; red line in **a**), and three axial planes (**d**, **e**, **f**; blue lines in **a**) predefined as reference for visual and quantitative assessment

Phantom measurements were performed using three identically shaped femoral components from total hip arthroplasty: Müller Straight Stem (size 10, length 140 mm, width 12.6 mm, offset 40.8 mm, neck length 39.4 mm) made of three different alloys: titanium (Ti), cobalt-chromium (Co-Cr), and stainless steel (Fe-Cr). Implants were bedded on a foam padding (polyurethane) in a water phantom (Fig. 1a). Identical placement of all femoral components within the water phantom for each scan was ensured through markers drawn on the foam padding.

### MRI protocol

Imaging was performed on three different systems from the same manufacturer (Siemens Healthineers, Erlangen, Germany): a 0.55-T Magnetom Free.Max; a 1.5-T Magnetom Avanto FIT; a 3-T Magnetom Prisma. Technical specifications of scanners are summarized in Table 1.

**Table 1:** Specifications of 0.55-T, 1.5-T, and 3-T scanner systems

	Bore width (cm)	Field strength (T)	Gradient amplitude (mT/m)	Slew rate (T/m/s)
Magnetom Free.Max	80	0.55	26	45
Magnetom Avanto FIT	60	1.50	45	200
Magnetom Prisma	60	3.00	80	200

Each femoral component was scanned separately using an optimized imaging protocol with metal artifact reduction sequences (MARS) from clinical routine. Pulse sequences included coronal T2-weighted turbo spin-echo (TSE), coronal turbo inversion-recovery magnitude (TIRM), axial T1-weighted TSE, axial TIRM, and sagittal T1-weighted TSE. Pulse sequence parameters are summarized in Table 2. Six-channel receiving flex coils were used in combination with receiving spine coils integrated in the scanners’ patient tables.

**Table 2:** Imaging protocols and pulse sequence parameters for 0.55-T, 1.5-T and 3-T scanner systems

Field strength	Plane	Sequence	TR (ms)	TE (ms)	TI (ms)	BW (Hz/pixel)	FOV (mm)	Matrix	ST (mm)	TA (min)
0.55 T	Coronal	T2 TSE TIRM	4,440	35	100	299	220 × 220	134 × 192	3.5	02:48
	Coronal	T2 TSE	3,220	77	-	434	220 × 220	224 × 320	3.5	02:21
	Axial	T2 TSE TIRM	4,260	25	100	302	220 × 220	192 × 240	5	04:58
	Axial	T1 TSE	517	9.4	-	401	220 × 220	213 × 304	5	03:40
	Sagittal	T1 TSE	517	9.4	-	401	220 × 220	213 × 304	5	03:40
1.5 T	Coronal	T2 TSE TIRM	4,000	34	150	455	220 × 220	192 × 256	3.5	01:48
	Coronal	T2 TSE	3,400	73	-	430	220 × 220	358 × 448	3.5	01:25
	Axial	T2 TSE TIRM	5,210	54	150	400	180 × 180	224 × 320	5	03:33
	Axial	T1 TSE	661	9.5	-	305	200 × 200	218 × 256	5	05:44
	Sagittal	T1 TSE	596	8.5	-	460	220 × 220	256 × 320	5	02:32
3 T	Coronal	T2 TSE TIRM	4,800	42	210	505	240 × 240	240 × 320	3.5	01:36
	Coronal	T2 TSE	4,500	73	-	505	220 × 220	224 × 320	3.5	01:30
	Axial	T2 TSE TIRM	4,200	39	200	500	180 × 180	205 × 256	5	02:06
	Axial	T1 TSE	687	17	-	520	200 × 200	320 × 320	5	02:53
	Sagittal	T1 TSE	600	9.3	-	520	240 × 240	384 × 384	5	02:32

*BW* Bandwidth, *FOV* Field of view, *ST* Slice thickness, *T1* T1-weighted, *T2* T2-weighted, *TA* Acquisition time, *TE* Echo time, *TI* Inversion time, *TIRM* Turbo inversion-recovery magnitude, *TR* Repetition time, *TSE* Turbo spin-echo

### Visual and quantitative artifact assessment

Artifact severity was visually assessed on all pulse sequences at two time points by a fellowship-trained musculoskeletal radiologist with 8 years of experience, a musculoskeletal radiology fellow with 2 years of experience and an orthopedic surgeon with 19 years of experience in hip surgery. A 7-point Likert scale (0 = no artifacts, 1 = minimal artifacts, 2 = mild artifacts, 3 = moderate artifacts, 4 = severe artifacts, 5 = very severe artifacts, 6 = extremely severe artifacts) was used for assessment [16]. Readers were blinded to pulse sequences, field strengths, alloys, and other readers’ assessments. Four weeks were allotted between the two reading sessions to reduce recall bias. The order of the pulse sequences was varied by random computer sorting for the second reading, and each reader was blinded to its prior interpretation.

Visual assessment of artifact severity was performed on nine slices for each implant at each field strength: one slice at the center of the femoral component for both coronal acquisitions (coronal T2-weighted TSE and T2-weighted TSE TIRM; Fig. 1b), one slice at the center of the femoral component for the sagittal acquisition (sagittal T1-weighted TSE; Fig. 1c) and three slices located in the proximal, middle, and distal portion of the femoral component for the two axial acquisitions (axial T2-weighted TSE TIRM and T1-weighted TSE; Fig. 1d–f). In total, 81 slices (3 alloys × 3 field strengths × 9 slices) were assessed twice by each reader.

**Fig. 2:**
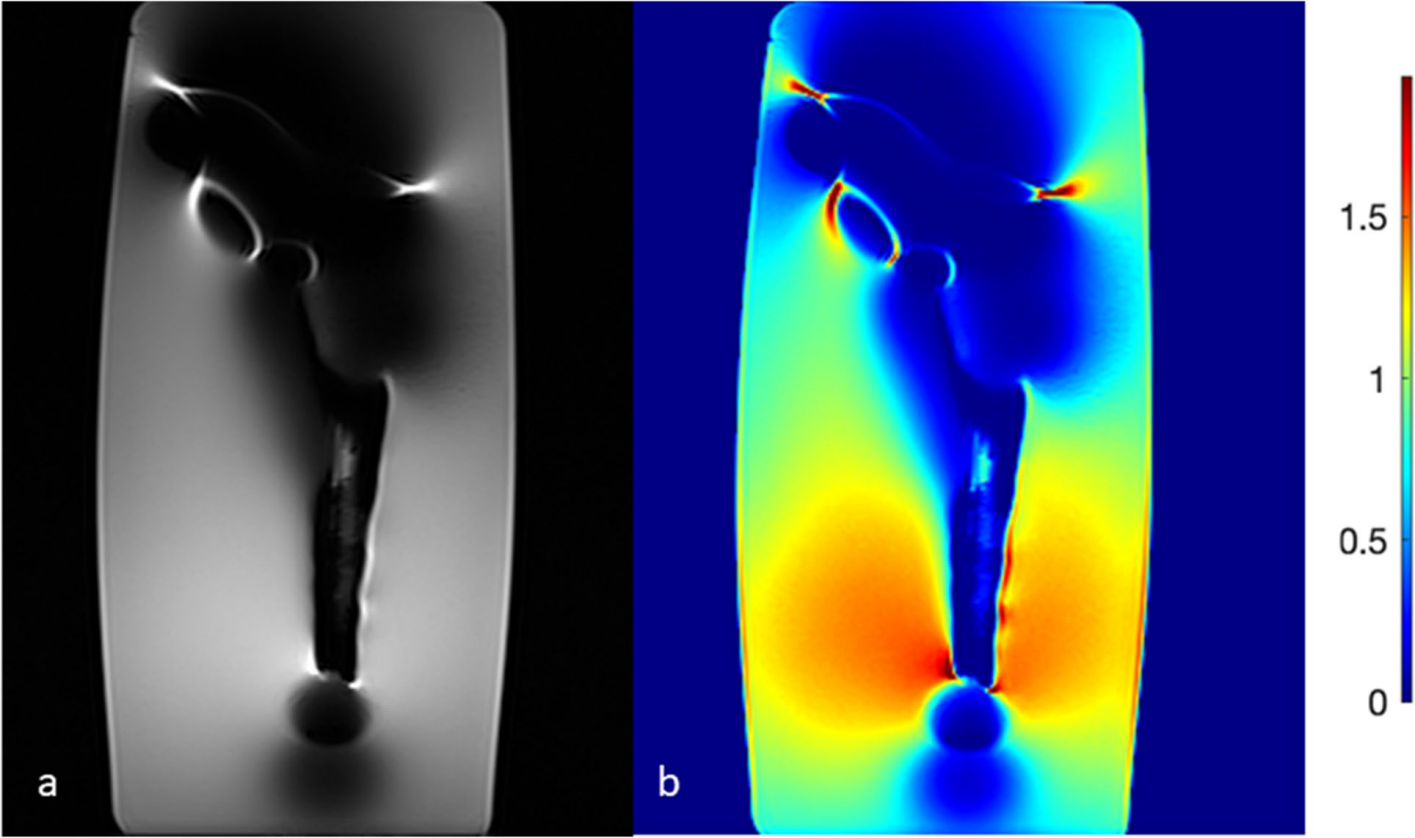
Coronal T2-weighted turbo spin-echo image of an implant (Fe-Cr) at 1.5 T (**a**) with the normalized signal intensity on the right (**b**)

**Fig. 3:**
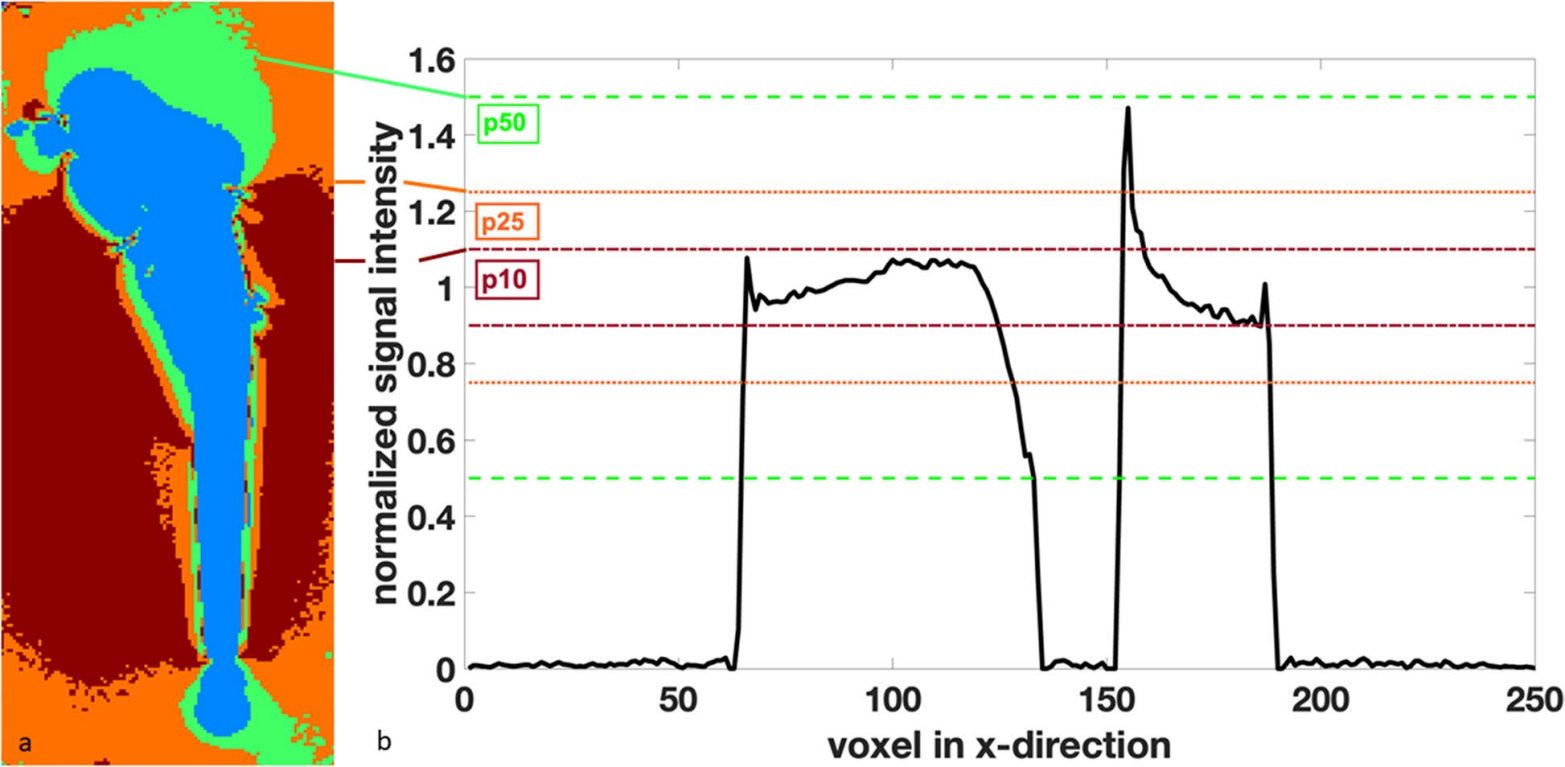
Scheme of the fractions of voxel within a 10%, 25%, and 50% limit above or below the mean signal intensity (**a**). Corresponding signal curve along the *x*-axis at the middle of the shaft with visualization of the fractions p10, p25, and p50 (**b**). See text (in the “Methods” section) for explanation of p10, p25, and p50

Quantitative assessment of artifact severity was performed on the same nine slices per implant and field strength through calculation of the coefficient of variation. Furthermore, a normalized signal intensity map was calculated for each slice (Fig. 2) and the fractions of voxels within the water phantom lying 10% (p10), 25% (p25), and 50% (p50) above or below the mean signal intensity of an automatically defined control region of interest were determined (Fig. 3).

### Statistical analysis

Interreader and intrareader agreement were determined by interclass and intraclass correlation coefficient (ICC). ICC values were interpreted as poor (ICC < 0.40), fair (ICC = 0.40–0.59), good (ICC = 0.60–0.74), and excellent (ICC = 0.75–1.0) [17]. Statistical differences between Likert scores were evaluated using the Wilcoxon signed-rank test. Correlation between Likert scores from visual assessment and parameters from quantitative assessment was determined using Spearman’s rho. Coefficient values were categorized as weak (*r* ≤ 0.30), moderate (*r* = 0.31–0.69), and strong (*r* = 0.70–1.0) [18]. *p* values < 0.05 were considered to represent a statistically significant difference.

## Results

### Intrareader and interreader agreement

**Fig. 4:**
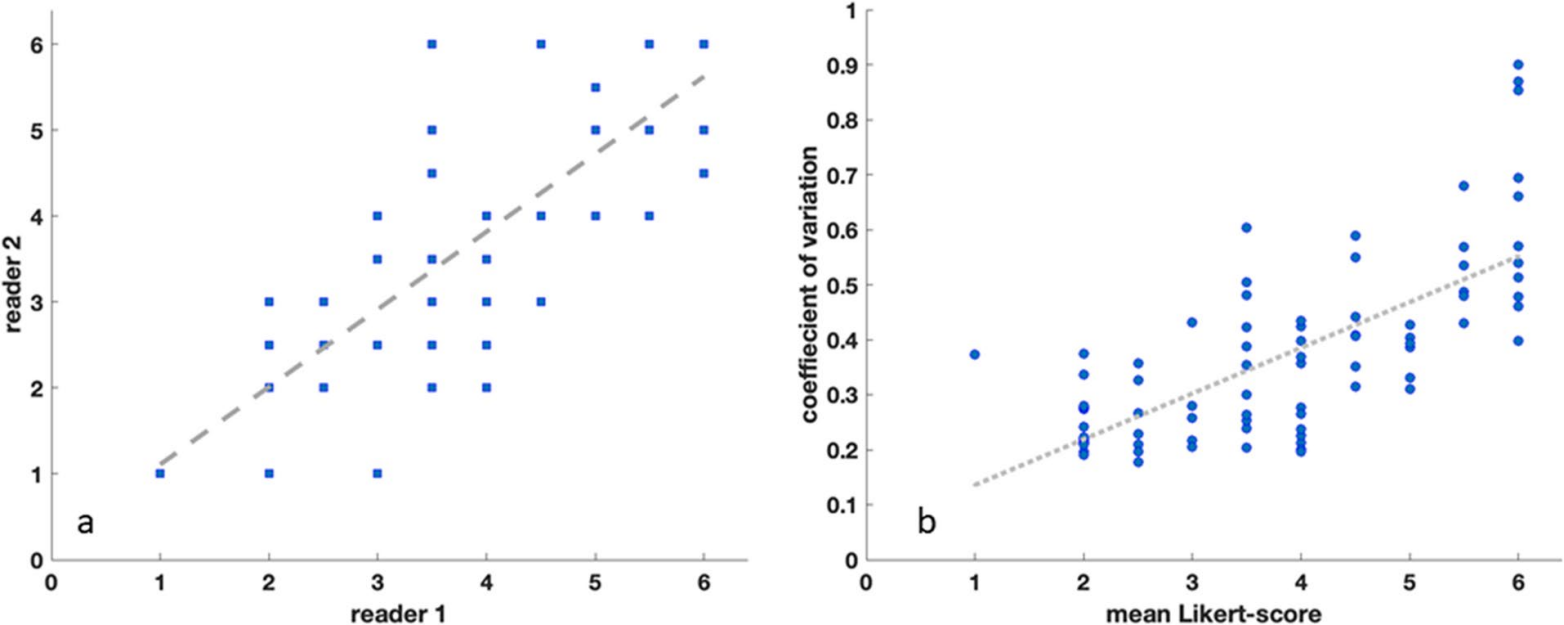
Scatter plots showing the correlation of reader 1 and 2 (**a**) and the correlation of mean Likert scores and coefficients of variation (**b**)

Intrareader agreement between the two reading sessions was excellent for all three readers (reader 1, ICC 0.89; reader 2, ICC 0.92; reader 3, ICC 0.78). The interreader agreement between the two radiologists (reading 1, ICC 0.76; reading 2, ICC 0.82; overall. ICC = 0.82; Fig. 4a) and the interreader agreement between the two radiologists and the orthopedic surgeon (reading 1, 0.66−0.68; reading 2, 0.80−0.82; overall, ICC 0.74−0.77) were good to excellent.

### Visual artifact assessment

Artifact severity was scored lowest for the femoral component made of Ti at 0.55 T (mean Likert score: 2.46 ± 1.03). Likewise, artifact severities of the Ti implant were rated lower than those of the other alloys both at 1.5 T (2.91 ± 1.16 for Ti *versus* 3.93 ± 1.46 for Co-Cr and 4.11 ± 1.63 for Fe-Cr; *p* = 0.151 and *p* = 0.091, respectively) and at 3 T (3.72 ± 0.91 for Ti *versus* 4.67 ± 1.05 for Co-Cr and 5.17 ± 1.06 for Fe-Cr; *p* = 0.060 and *p* < 0.001, respectively).

Artifact severities of all three alloys were scored significantly lower at 0.55 T compared to 3 T (*e.g.*, Ti implant 2.46 ± 1.03 *versus* 3.72 ± 0.91, *p* < 0.001). Also at 1.5 T, the artifact severity scores determined by the three readers were significantly lower for Ti than those at 3 T (*e.g.*, Ti implant 2.91 ± 1.16 *versus* 3.72 ± 0.91, *p* = 0.043). Visual assessment however did not reveal statistically significant differences between artifact severities at 0.55 T and 1.5 T (*e.g.*, Ti implant 2.46 ± 1.03 *versus* 2.91 ± 1.16, *p* = 0.451).

While artifacts of the Co-Cr implant were scored slightly less severe than those of stainless steel implants at all three field strengths, the differences were not statistically significant (*e.g.*, at 3T, 4.67 ± 1.05 for Co-Cr *versus* 5.17 ± 1.06 for Fe-Cr, *p* = 0.254). All Likert scores and *p* values are listed in Table 3.

**Table 3:** Likert scores overall and per reader for each distinct alloy at 0.55T, 1.5T, and 3T

		Reader 1	Reader 2	Reader 3	Overall	Significant *p* values
0.55 T	Titanium	2.33 ± 0.90	1.89 ± 0.83	3.17 ± 1.10	2.46 ± 1.03 (*,**,+)	*: *p *< 0.01 +: *p *= 0.08 ++: *p *= 0.49 +++: *p *= 0.042 −*: p *= 0.042 o: *p *= 0.07
	Cobalt-chromium	3.67 ± 0.43	3.94 ± 0.85	4.44 ± 0.92	4.02 ± 0.51 (*,++)	
	Stainless steel	4.33 ± 0.94	3.50 ± 1.97	4.78 ± 0.87	4.20 ± 1.11 (**,+++)	
1.5 T	Titanium	2.78 ± 1.15	2.94 ± 1.13	3.00 ± 1.44	2.91 ± 1.16 (−)	
	Cobalt-chromium	3.83 ± 1.44	4.00 ± 1.48	3.94 ± 1.65	3.93 ± 1.46	
	Stainless steel	3.94 ± 1.79	4.22 ± 1.56	4.17 ± 1.64	4.11 ± 1.63	
3 T	Titanium	4.00 ± 1.12	3.17 ± 1.09	4.00 ± 1.12	3.72 ± 0.91 (o,+,−)	
	Cobalt-chromium	4.61 ± 1.14	4.56 ± 1.36	4.83 ± 1.12	4.67 ± 1.05 (+,++)	
	Stainless steel	5.22 ± 1.00	5.00 ± 1.32	5.28 ± 0.97	5.17 ± 1.06 (o,+,+++)	

Data are given as mean ± standard deviation. Pairwise significant differences of the mean score existed between titanium at 0.55 T and cobalt-chromium/stainless steel at 0.55 T (*/**), titanium at 0.55 T and titanium at 3 T (+), titanium at 1.5 T and titanium at 3 T (−), cobalt-chromium at 0.55 T and cobalt-chromium at 3 T (++), stainless steel at 0.55 T and stainless steel at 3 T (+++), and between Titanium at 3 T and stainless steel at 3 T (−)

### Quantitative artifact assessment

The lowest artifact intensities were measured for the femoral component made of Ti at 1.5 T and 0.55 T with a coefficient of variation of 0.28 ± 0.08 and 0.31 ± 0.06, respectively. Similarly, quantitative artifact assessment showed no statistically significant differences for the two other alloys at 0.55 T and 1.5 T: Co-Cr 0.35 ± 0.09 *versus* 0.35 ± 0.10 and Fe-Cr 0.37 ± 0.12 *versus* 0.36 ± 0.16 (*p* ≥ 0.059).

Strongest artifacts were observed for the femoral component made of stainless steel at 3 T with a coefficient of variation of 0.62 ± 0.19. The fraction of voxels lying within a threshold of 10%, 25%, and 50% above or below mean signal intensity were 0.25 ± 0.15, 0.47 ± 0.20, and 0.64 ± 0.20, respectively.

**Fig. 5:**
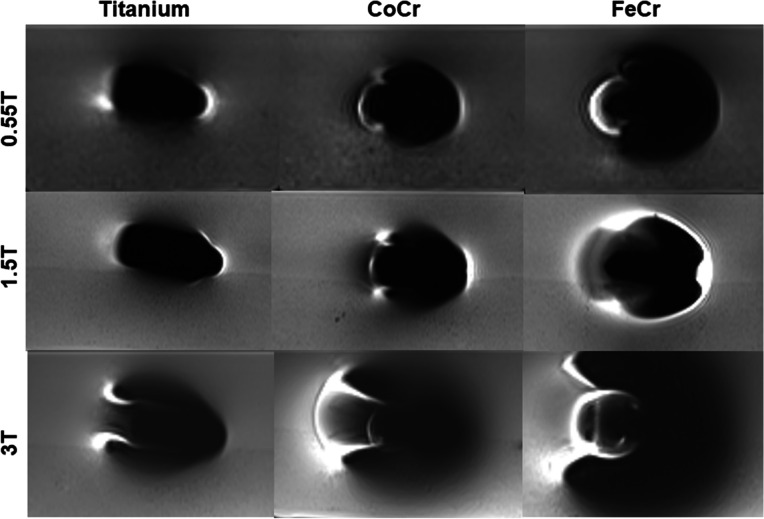
Axial slices through the femoral components made of three different alloys at 0.55 T, 1.5 T, and 3 T. Artifacts are lowest for Ti and increase with higher magnetic field strength for all three alloys

**Fig. 6:**
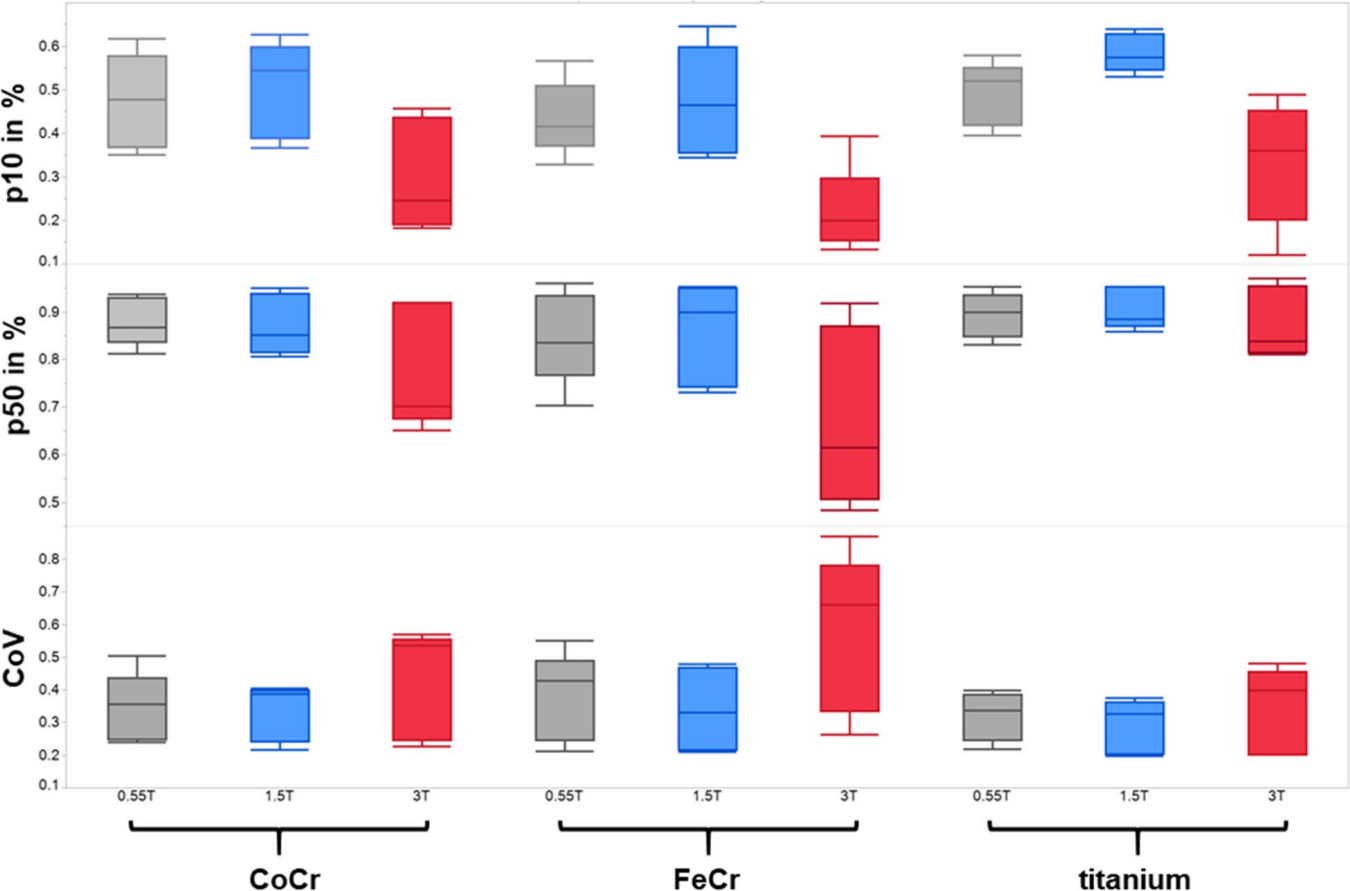
Overview of the quantitative parameters p10, p50, and coefficients of variation (CoV) for the different field strengths and alloys. See text (in the “Methods” section) for explanation of p10, p25, and p50

Artifacts were less severe for Ti and Co-Cr alloys than for implants made of stainless steel at all three field strengths (Table 4, Figs. 5 and 6).

**Table 4:** Coefficient of variation and fraction of voxels within a 10% (p10), 25% (p25), and 50% (p50) limit above or below mean signal intensity within the phantom at 0.55 T, 1.5 T, and 3 T. No statistically significant differences exist

		CoV	p10	p25	p50
0.55 T	Titanium	0.31 ± 0.06	0.41 ± 0.11	0.74 ± 0.10	0.90 ± 0.04
	Cobalt-chromium	0.35 ± 0.09	0.37 ± 0.15	0.69 ± 0.14	0.86 ± 0.06
	Stainless steel	0.37 ± 0.12	0.38 ± 0.10	0.69 ± 0.11	0.85 ± 0.09
1.5 T	Titanium	0.28 ± 0.08	0.55 ± 0.07	0.82 ± 0.05	0.91 ± 0.04
	Cobalt-chromium	0.35 ± 0.10	0.46 ± 0.16	0.73 ± 0.14	0.85 ± 0.09
	Stainless steel	0.36 ± 0.16	0.44 ± 0.16	0.70 ± 0.19	0.83 ± 0.14
3 T	Titanium	0.34 ± 0.13	0.30 ± 0.13	0.66 ± 0.18	0.86 ± 0.12
	Cobalt-chromium	0.43 ± 0.19	0.31 ± 0.16	0.59 ± 0.24	0.75 ± 0.21
	Stainless steel	0.62 ± 0.19	0.25 ± 0.15	0.47 ± 0.24	0.64 ± 0.20

*CoV* Coefficient of variation

### Correlation between visual and quantitative assessment

There was a strong positive linear correlation between the calculated coefficients of variation and the three readers’ Likert scores from visual artifact severity assessment (*r* = 0.87; *p* = 0.005, Fig. 4b). Strong negative linear correlations were seen for both the 25% and 50% thresholds of voxels lying above or below mean signal intensity and the Likert scores (p25, *r* = -0.84, *p*=0.005; p50, *r* = -0.85, *p* = 0.007). Moderate negative correlation was observed for p10 and visual assessments’ Likert scores (*r* = -0.73; *p* = 0.032)

## Discussion

The aim of our study was to visually and quantitatively evaluate the influence of field strength and implant alloys on susceptibility-induced artifacts in hip implant imaging. Although artifact severities were scored lowest for Ti implants at 0.55 T in terms of absolute values (mean Likert score: 2.46 ± 1.03), visual assessment showed no significant differences between imaging at 0.55 T and 1.5 T (*e.g.*, for Ti implants 2.46 ± 1.03 *versus* 2.91 ± 1.16, *p* = 0.451). Strong intrareader agreement (ICC 0.78−0.92) and strong to moderate interreader agreement (ICC 0.74−0.77) were found between the two subspecialized musculoskeletal radiologists and the orthopedic surgeon during visual assessment of image quality. Their ratings strongly correlated with the parameters used for quantitative evaluation of artifact intensities, *i.e.*, with the coefficient of variation (*r* = 0.87; *p*=0.005). To our best knowledge, this is the first study which has been performed on a commercially available, non-experimental 0.55-T scanner.

Both visually and quantitatively, artifacts of Ti alloys were least severe, while those of stainless steel implants were strongest at all three field strengths. This is in accordance with recently published literature [12, 19]. Artifacts for all three alloys were rated lower at 0.55 T and 1.5 T compared to 3 T during visual assessment. Although artifacts were evaluated slightly less severe at 0.55 T compared to 1.5 T for some alloys (*e.g.*, for Ti 2.46 ± 1.03 *versus* 2.91 ± 1.16), these differences were not statistically significant.

During quantitative artifact assessment, the lowest artifact intensities were observed for the Ti implant at 1.5 T. This contrasts previous investigations, *e.g.*, the study of Matsuura et al., who demonstrated less artifacts for neurosurgical biomaterials at 0.5 T compared to 1.5 T and 3 T [20, 21]. This discrepancy might be explained by substantial differences in technical specifications of the scanner systems used. Gradients of our commercially available 0.55-T scanner system were lower compared to the experimental 0.5-T scanner system used by Matsuura et al., as well as compared to clinically established 1.5-T and 3-T scanner systems (Table 1). Furthermore, our 0.55-T system operates with a substantially larger bore width of 80 cm (compared to 60 cm for the 1.5-T and 3-T scanner systems) to improve patient comfort [22].

Our study has several limitations. For imaging at 1.5 T and 3 T, we used optimized pulse sequences from clinical routine, which were not further amended for scanning at 0.55 T, as the primary aim of our investigation was to evaluated the influence of magnetic field strength alone. With addition optimization of pulse sequence parameters, susceptibility artifacts at 0.55 T might me further reduced. Especially the effects of advanced imaging techniques such as VAT or SEMAC, which are known to improve image quality at 1.5 and 3 T, have to be evaluated at 0.55T [15]. In addition to that, a phantom-based evaluation of susceptibility-induced artifacts only allows to forecast which artifacts might have to be expected with *in vivo* scanning. Investigations on how these artifacts actually impact the evaluation of distinct surrounding anatomic structures must include prospective scanner-scanner comparison studies in human subjects. Although a new objectifiable and reproducible method for artifact quantification was created, which has a good agreement with the subjective ratings, the influence of factors such as instrument-related specifications (for example, bore width or homogeneity of the B_0_ field) remains unclear. In particular, an increasing field causes a shortening wavelength and results in a nonuniformity of intensity profiles. Through the respective normalization to the image to be evaluated, an attempt was made to objectify this as best as possible. Finally, visual perception and evaluation of artifacts by the individual readers is highly subjective and might differ between radiologists and other clinicians. Therefore, visual assessment was performed both by two musculoskeletal radiologists and one orthopedic surgeon and quantitative parameters were used to correlate with visual evaluation.

In conclusion, our results demonstrate that susceptibility-induced artifacts of hip implants at a commercially available 0.55-T scanner are comparable to MARS imaging at 1.5 T despite an inferior gradient system and larger bore width. The additional use of advanced imaging techniques and reconstruction mechanisms [19, 23] may further reduce artifacts at 0.55 T. Current limitations of metal implant imaging at higher field strengths, *i.e.*, specific absorption rates and tissue heating [24, 25] might also be overcome with low field MRI at 0.55 T.

## Data Availability

The datasets used and/or analyzed during the current study are available from the corresponding author on reasonable request.

## Abbreviations

Co-Cr: Cobalt-chromium
Fe-Cr: Iron-chromium (stainless steel)
ICC: Intra/inter-class correlation coefficient
MRI: Magnetic resonance imaging
MARS: Metal artifact reduction sequences
p10: Fractions of voxels within the water phantom lying 10%
p25: Fractions of voxels within the water phantom lying 25%
p50: Fractions of voxels within the water phantom lying 50%
SEMAC: Slice encoding for metal artifact correction
Ti: Titanium
TIRM: Turbo inversion-recovery magnitude
TSE: Turbo spin-echo
VAT: View angle tilting
